# Cyclodextrins and Amino Acids Enhance Solubility and Tolerability of Retinoic Acid/Tretinoin: Molecular Docking, Physicochemical, Cytotoxicity, Scratch Assay, and Topical Gel Formulations Investigation

**DOI:** 10.3390/pharmaceutics16070853

**Published:** 2024-06-25

**Authors:** Zeinab Fathalla, Mai E. Shoman, Hebatallah S. Barakat, Adel Al Fatease, Ali H. Alamri, Hamdy Abdelkader

**Affiliations:** 1 Department of Pharmaceutics, Faculty of Pharmacy, Minia University, Minia 61519, Egypt; zianab.mohamed@minia.edu.eg; 2 Department of Medicinal Chemistry, Faculty of Pharmacy, Minia University, Minia 61519, Egypt; mai_shoman@mu.edu.eg; 3 Department of Pharmaceutics, Faculty of Pharmacy, Alexandria University, Alexandria 21525, Egypt; hebatallah.soliman@alexu.edu.eg; 4 Department of Pharmaceutics, College of Pharmacy, King Khalid University, Abha 62223, Saudi Arabia; afatease@kku.edu.sa (A.A.F.); aamri@kku.edu.sa (A.H.A.)

**Keywords:** retinoic acid, l-arginine, cyclodextrins, aging, cytotoxicity, scratch assay, gels

## Abstract

With increasing longevity globally, the search for effective and patient-friendly anti-aging solutions has been growing. Retinoic acid (Ret) is an FDA-approved anti-aging and anti-wrinkling formula, however, its poor solubility and poor tolerability hamper its use in cosmetically accepted formulations. In this study, cyclodextrins and arginine were investigated for improving the solubility and tolerability of retinoic acid through the formation of inclusion complexes and salt formation, respectively. Two different methods were employed: physical mixing and kneading. The prepared dispersions were investigated for molecular docking (MD), solubility, thermal and spectral analyses, cytotoxicity, and scratch assays. The optimized disperse systems were formulated in a gel formulation and characterized for rheological, in vitro release, and kinetics. The MD, DSC, and FTIR results indicated that both β- and hydroxy propyl (HP) β-cyclodextrins could host RA in their cavities and form inclusion complexes. Ret can form a salt with the basic amino acid arginine. Solubility studies of RA significantly (*p* < 0.01) enhanced by 14- to 81-fold increases with the investigated cyclodextrins and arginine. The cell viability recorded for Ret:HP β-CD K and Ret:arginine K was significantly increased compared to that for Ret alone. The IC50% recorded for azelaic acid (mild to non-irritant control), Ret, Ret:HP β-CD K, and Ret:arginine K were 1000, 485, 1100, and 895 µg/mL, respectively. The two carriers (HP β-CD and the amino acid arginine) were able to significantly (*p* < 0.05) reduce the irritation potential of Ret. Furthermore, comparable gap closure rates were recorded for Ret alone, Ret:HP β-CD K, and Ret:arginine K, indicating that inclusion complexation and ion pair formation reduced the irritation potentials without undermining the efficacy.

## 1. Introduction

**Figure 1:**
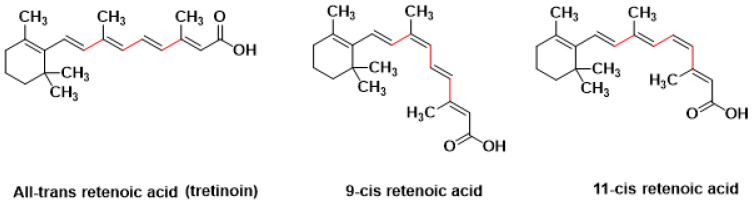
Chemical structures and different geometrical isomers of retinoic acid.

The active form of vitamin A is generically known as tretinoin and retinoic acid (Figure 1). It is widely used commercially in the form of creams for the treatment of mild to moderate acne vulgaris [1]. Retinoic acid is the most effective but least tolerable form of retinoids, which includes various chemical forms such as retinyl ester, retinol, retinaldehyde, and retinoic acid [2]. Acne vulgaris is a very common chronic skin problem affecting young people aged 13 to 24 years old [3]. Recent reports suggest retinoic acid is a very effective anti-aging drug [2]. Retinoic acid acts as keratolytic and reduces sebum production by binding to retinoic acid receptors; hence, it helps to remove comedones and eases follicular occlusion [4]. Tretinoin regulates cell apoptosis, differentiation, and proliferation [5]. Further benefits can be obtained from topical retinoic acid administration, including treatment of photodamaged skin and skin aging [1]. Tretinoin can be considered the most effective and has proven anti-wrinkle and anti-aging treatment [2].

Retinoic acid is an extremely hydrophobic drug and practically insoluble in water [3]. Poor solubility poses challenges to its formulation development. This could limit the formulation of tretinoin to conventional creams [3,6]. Another limitation related to retinoic acid is a condition known as retinoic dermatitis. This retinoic acid syndrome includes skin dryness, photosensitivity, skin exfoliation, irritation, a burning sensation, and redness [7]. Conventional creams are not cosmetically accepted with repeated modes of administration. Creams have a greasy appearance, which makes them inconvenient. In addition to poor solubility, poor stability, skin irritation, and poor compliance pose further challenges toward tretinoin delivery in appropriate dosage forms [2]. For these reasons, there is a compelling need to develop novel formulations for retinoic acid with improved safety, reduced irritation, and advanced water solubility [6].

Liposomes and proniosomes encapsulation of retinoic acid has been reported to increase skin tolerability and safety [3,8]. Nevertheless, retinoic acid liposomes and proniosomes are yet to be commercially available due to the poor physical and chemical stability of liposomes.

Cyclodextrins (CDs) are a group of three nature (α-, β- and γ-CDs) and numerous derivatives of cyclic sugar moieties that form trojan horse-like domains to hydrophobic drugs. Hence, the solubility, stability, tolerability, and permeability of those drugs that fit into the cavities of CDs can be improved [9]. CDs have been used in many pharmaceutical products (>30 pharmaceuticals) for oral, injectable, and ophthalmic drug delivery [10,11,12,13,14]. In cosmetic and dermatological pharmaceutical products, CDs have still been gaining popularity. CDs were investigated for skin drug delivery without altering the skin structure and showed high safety and tolerability for dermal and transdermal drug delivery [15].

For example, CDs were included in sunscreen products to improve their stability, protect sunscreen agents from sunlight-induced rapid degradation, and hence prolong the activities of the sunscreen products [9]. Such useful properties advance CDs as functional excipients. We hypothesized that CDs could play a pivotal role in improving solubility and reducing the irritation potential of retinoic acid. This study aimed to study the formation of RA:CDs inclusion complexes and evaluate these complexes for solubility, dissolution, in vitro irritation studies (e.g., the MTT assay) and scratch wound assays.

## 2. Materials and Methods

Retinoic acid was purchased from TCI Chemicals, Tokyo, Japan. β-cyclodextrin (β-CD) and hydroxypropyl β-cyclodextrin (HP β-CD) were purchased from Acros Organics, Bridgewater, NJ, USA. L-arginine and carboxymethyl cellulose were supplied by Fluka AG, Buchs, Switzerland. Pluronic F127, Tween 80, and cellulose membrane (with a molecular weight cut-off of 12–14 KDa) were purchased from Sigma-Aldrich, London, UK. Gellan gum was supplied by Loba Chemie, Mumbai, India. Avtotin-A cream (0.05% tretinoin acid) was provided by Avalon Pharma, Riyadh, Saudi Arabia.

### 2.1. Preparation of Retinoic Acid-Additives Physical and Kneading Dispersions

Individual weights of RA, L- arginine, β-CD, and HP β-CD were accurately weighed in the equivalent of molar ratios in milligrams (mg). Every drug/additive was mixed thoroughly in a plastic Petri dish using a spatula until a homogenous color was obtained (Figure 1). The physical mixtures produced were transferred into Eppendorf tubes and stored in a dry, cool place until further use.

For preparation of the kneaded mixtures, additional batches of the above physical mixtures were prepared, and a hydroalcoholic solution (50% *v*/*v*) was dropped into the mixtures and mixed well till a thick and malleable dough formed. The dough was dispersed on the Petri dish and allowed to air dry. The dried mixture was pulverized using a pestle, transferred into Eppendorf tubes, and stored in a cool, dry place until further use.

### 2.2. Molecular Docking

Molecular Operating Environment (MOE) version 2014.09 software (Chemical Computing Group, Montreal, QC, Canada) was used to visualize the RA:additive possible binding sites and estimate the binding constants.

### 2.3. Equilibrium Solubility Studies

Excess amounts of RA, RA:additive PM, and RA:additive K mixtures were transferred into glass vials (20 mL capacity) containing 10 mL of distilled water. The stoppered glass vials were placed in a thermostatic shaking water bath (Shel Lab water bath, Sheldon Cornelius, Cornelius, OR, USA) at 37 °C ± 0.5 °C at a speed of 120 strokes per min. The samples were left for 48 h; aliquots were withdrawn, filtered, rendered alkaline, and determined at 340 nm using the Shimadzu UV-2550 spectrophotometer, Kyoto, Japan, at 362 nm.

### 2.4. Differential Scanning Calorimetry (DSC)

Specific weights of the RA, RA:additive PM, and RA:additive K mixtures were placed in a 40 µL-capacity aluminum pan. The pan temperature was allowed to increase from 30 °C to 300 °C at 10 °C/minute using a DSC calorimeter (PerkinElmer DSC 4000, Hoogvliet, The Netherlands) and Pyris Manger Properties software (version 11). Nitrogen was used as a purging gas at a flow rate of 20 mL/minute.

### 2.5. Fourier Transform Infrared Spectroscopic (FTIR) Study

FTIR spectrophotometry (Agilent Cary 360 FTIR, Agilent Technologies, Jaya, Malaysia) and MicroLab FTIR software (version 5.7) were used to stack the spectra for the above-mentioned samples in Section 2.5. The spectra were collected directly from the dispersed powder on the diamond surface. The spectra were collected in a range of 4000 to 400 cm^−1^.

### 2.6. Cytotoxicity Assay

In vitro irritation studies on RA, RA:arginine K, and RA:HP β-CD K using the cytotoxicity assay were conducted. The human fibroblast (hFB-4) cells were obtained from Vacsera Co., Giza, Egypt. A 96-well plate was inoculated with 1 × 10^5^ cells/mL (100 μL/well) and incubated at 37 °C for 24 h for confluency. The RPMI medium with 2% serum was discarded, and the cells were washed twice with the media. Serial dilutions of the samples and the negative control (azelaic acid) were made using the growth media. An aliquot of 0.1 mL of each sample and control was tested in different wells, leaving 3 wells as controls, receiving only a maintenance medium. The MTT solution was prepared (5 mg/mL in PBS) (Biobasic Inc., Markham, ON, Canada). A volume of 20 µL MTT solution was added to each well, placed on a shaker at 150 rpm for 5 min, and then incubated (37 °C, 5% CO_2_) for 4 h. The media were discarded, and formazan (MTT metabolic product) was resuspended in 200 μL of DMSO. The optical density was measured at 560 nm and a subtracted background at 620 nm.

### 2.7. Scratch Assay

Six-well plates were used to seed the hFB-4 cells, which were then grown to confluence. To create a wound, the bottom of the plate was scratched with a yellow pipette tip. To keep the scratch breadth within a certain range, the pipette tip was angled at about thirty degrees. This made it possible to image both wound edges with the conventional light microscope’s 10× objective. The wound healing parameters were estimated as follows [16]:

$$\mathrm{Rate}\;\mathrm{of}\;\mathrm{migration}\;(\mathrm{RM})=\frac{Wi-Wf}{t}$$

where Wi and Wf are the initial and ultimate wound widths in µm, and t is the period of the assay in hours.

$$\mathrm{Wound}\;\mathrm{closure}\;\%=\frac{Ao-At}{Ao}\times 100$$

where Ao and At are the wound area (µm^2^) at t = 0 and t = measured time points, respectively.

Area difference % = Ai − A(Ai − Af)

Ai = initial area

Af = final area

### 2.8. Gel Preparation

Five different gel formulations were prepared from three different polymers as follows: Pluronic F127, carboxymethyl cellulose (CMC), and gellan gum, according to the composition shown in Table 1. In brief, accurately weighed amounts of gellan gum and CMC were dissolved in distilled water and stirred on a hot plate at 80 °C for 2 h. Pluronic F127 was dissolved in cold distilled water overnight. Specific amounts of Ret, Ret:arginine K, and RA:HP β-CD K were dispersed in the formed gels to form the final formulations F1–F5.

### 2.9. Viscosity Measurements

The viscosities of the retinoic acid-loaded gels were determined using a rotary viscometer (Selecta ST2001, Barcelona, Spain) connected to a spindle L2 type. The viscosity of the prepared samples was recorded at shearing rates from 5 to 100 rpm in ambient conditions.

### 2.10. Spreading Coefficients

A spreadability test was used to evaluate how much of the afflicted skin areas the topical application spreads to. A sample (1 g) of the formulations was applied in the center of a circle of 2 cm diameter on a plastic sheet. Another plastic sheet was placed and compressed to uniform thickness by placing 1000 gm of weight for 2 min. The spreadability (S) was calculated using the following formula [17]:

$$S=\frac{L}{2}\times 100$$

where S denotes spreadability, and L is the ultimate diameter (in cm) of the spread gel moved on the plastic slide.

### 2.11. In Vitro Release Study

An in vitro release study was performed using the modified Franze Diffusion cells method [18]. The release medium was composed of phosphate buffer with a pH of 6.8 (50 mL) containing 0.1% Tween 80 to the ensuing conditions. The donor compartment contained 1 g of gel formulations or Avtotin-A cream (0.05% retinoic acid), and the cellulose membrane (presoaked in the release medium) was spread between the donor and receptor compartments. The media were placed in a shaking water bath set at 37 °C and shaken at 100 rpm. A five-milliliter sample was taken out and replaced with five milliliters of the fresh-release medium. The samples were determined spectrophotometrically, as mentioned above in Section 2.4. The cumulative release data were fitted into the Korsmeyer–Peppas equation as below [19]:

$$\frac{Mt}{M\infty }=kt^{n}$$

where Mt/M∞ is the cumulative percentage released at time (t), k is the kinetic constant, and n is the release exponent.

### 2.12. Statistical Analysis

A two-way ANOVA and unpaired *t*-test were performed using Graph Pad Prism 8.4.3.686 for the statistical analysis of the results, with a significance level of *p* < 0.05.

## 3. Results and Discussion

**Figure 2:**
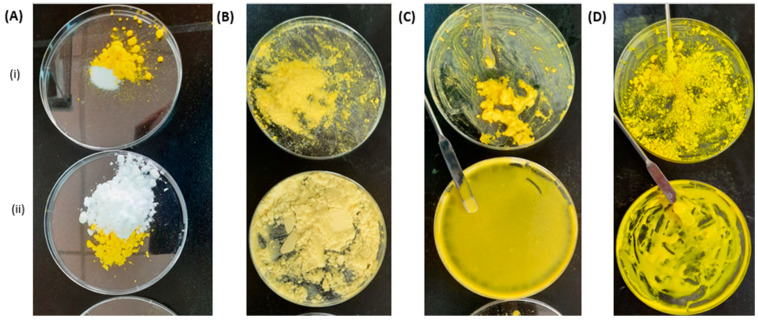
(**A**–**D**) Pictorial outline for RA:CDs complexes using physical and kneading methods. Yellowish brown powder and white powder represent RA and CD, respectively (**A**); homogenous physical mixtures for both β-CD (**i**) and HP β-CD (**ii**) formed (**B**); kneading the mixtures with a spatula using hydroalcoholic solution (50% *v*/*v*) (**C**); finally, dried, kneaded mixtures, read to grind (**D**).

One natural β-cyclodextrin and a derivative with the hydrophilic hydroxypropyl substitutions were investigated to form host–guest complexes with RA. Two techniques— the physical mixing and kneading techniques—were employed to form the solid dispersions. Figure 2A–D shows pictorial outlines for the different steps of physical mixing and kneading to prepare the RA:CDs-dispersed mixtures for further study and characterization. It is worth mentioning that the RA:HP β-CD-kneaded mixtures were well dissolved and dispersed in the Petri dish using the same amount of kneading solution, as demonstrated in Figure 2(C-ii). This could be ascribed to the greater solubility and hydrophilicity of the HP-β-CD derivative used compared to the less soluble native β-CD.

### 3.1. Molecular Docking

**Figure 3:**
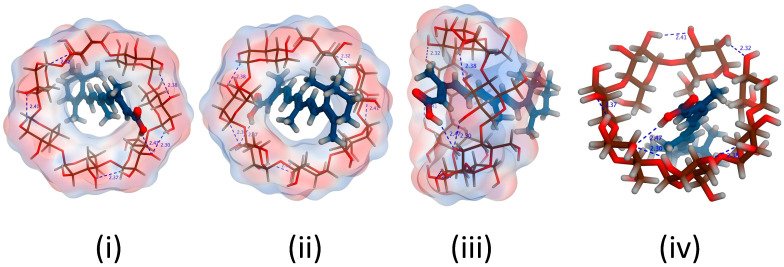
3D poses for Retinoic acid docked into the inclusion pocket of β-cyclodextrin showing (**i**) top view, (**ii**) bottom view (**iii**) side view (**iv**) top view with marked interactions demonstrating bond length.

**Figure 4:**
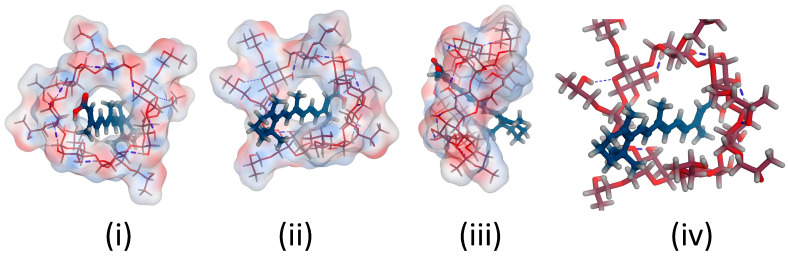
3D poses for Retinoic acid docked into the inclusion pocket of Hydroxypropyl-β-cyclodextrin showing (**i**) top view, (**ii**) bottom view (**iii**) side view (**iv**) top view with marked interactions demonstrating bond length.

The retinoic acid/cyclodextrin inclusion complexes’ stability was supported via molecular docking simulations. The results showed that stable complexes with energy of −5.9 and −5.0 Kcal/mol are theoretically formed upon solubilizing retinoic acid with both hydroxy propyl-β-cyclodextrin and β-cyclodextrin, respectively. The small diameter of the conjugated carbon perfectly fits the inclusion pocket for the two cyclodextrins used (Figure 3 and Figure 4), with the carboxylic acid tail offering a single position for hydrogen bond formation. Single H-bond formation was detected with both cyclodextrins, contributing to the higher stability of the complexes formed. Nevertheless, a shorter bond length and, hence, higher stability was detected with hydroxypropyl-β-cyclodextrin (1.77 A, energy of −5.7 Kcal/mol) and offers a potential explanation for the increased solubility observed empirically with hydroxypropyl β-cyclodextrin. Nevertheless, the absence of the hydroxypropyl groups in β-cyclodextrin might have barred the hydrophilic group from going further into the cyclodextrin pocket to adjust the carboxylic tail in the proper spot for such H-bonding construction and also resulted in a slightly decreased bond strength (bond length 2.47 A) and lower stability of the complex formed (energy −5.04 Kcal/mol) and, hence, explains the inferior solubility observed experimentally with β-cyclodextrin.

**Figure 5:**
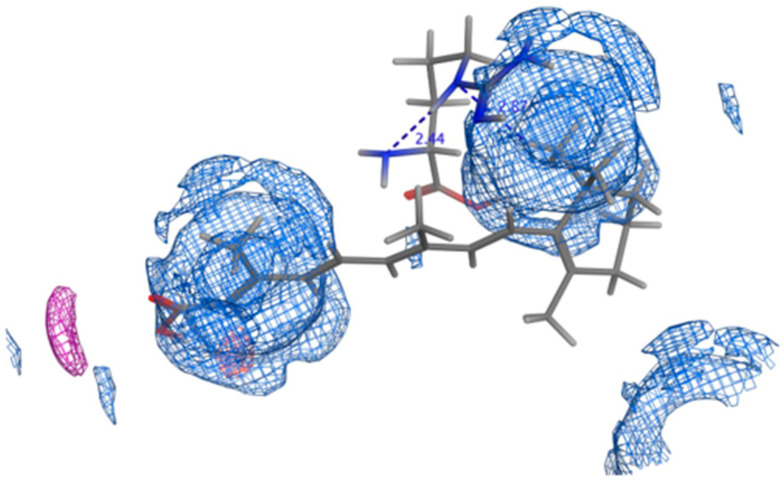
Predicted potential interactions between retinoic acid and arginine, showing an electrostatic map of retinoic acid’s surface, highlighting major areas of interaction.

The docking studies utilized the lipophilic head as a possible site of interaction with arginine, with the terminal guanidine group forming a H-bond with the electron-rich lipophilic head in a stable complex with an energy score of −3.6132 Kcal/mol (Figure 5). Additionally, the theoretical calculations implemented supported a salt formation between retinoic acid and arginine, explaining the fast solubilization process that occurred experimentally. The salt formation is hypothesized to be due to a difference greater than 3 between the pKa of arginine and retinoic acid. A difference between [pKa(base)-pKa(acid)] of greater than 3 is repeatedly associated with salt formation [20], with the current case pKa (base) of arginine being 12.48, while the calculated pKa (acid) of retinoic acid being around 5 strongly supports a soluble salt formation upon dissolution of both components together.

### 3.2. Solubility Studies

**Figure 6:**
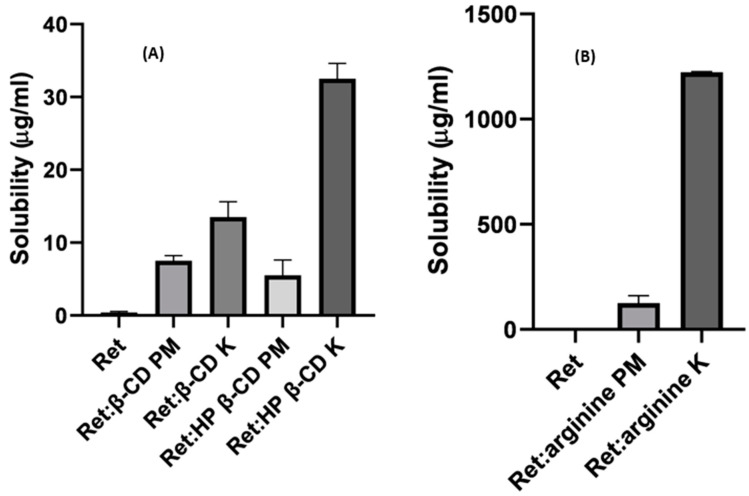
Solubility of retinoic (Ret) alone and Ret physical (PM) and kneading (K) mixtures with the two cyclodextrins (**A**) and arginine (**B**). Data represent means ± SD, n = 3.

The equilibrium solubilities of retinoic acid (Ret) alone and Ret from the prepared physical and kneaded mixtures in water were studied, and the results are shown in Figure 6A. The solubility of Ret recorded an extremely low value of <1 µg/mL. The solubility of Ret from the physical and kneaded mixtures with the two cyclodextrins recorded significant increases (*p* < 0.05) of 5.5 to 32.5 µg/mL. The solubility enhancement factors ranged from 14- to 81-fold increases (Figure 6A). The results indicated that the solubility of Ret was dependent on both the method of preparation and the type of cyclodextrins. For example, the solubility of Ret from Ret:β-CD PM and Ret:β-CD K was 7.5 and 13.5 µg/mL, respectively. The solubility of Ret from Ret:HP β-CD K was greater by 5.9-fold compared to that from Ret:HP β-CD K.

Similarly, the type of CDs recorded had a significant (*p* < 0.05) positive effect on the solubility of Ret. The solubility of Ret from Ret:HP β-CD K was greater by 2.4-folds, compared to that from Ret:β-CD K.

The kneading method involved the use of a common solvent system that could allow the dissolution of the drug and CDs at the molecular dispersion and enable more intimate interactions between the drug and CDs than physical mixing on the granular levels of particles. This explanation can be visualized in Figure 2, indicating that the kneading technique was superior compared to physical mixing as far as the solubility was concerned.

These results correlated well with those obtained from the molecular docking studies; HP β-CD had a stronger binding and was better fitting with Ret compared to β-CD, and this could explain the greater solubility of Ret from the Ret:HP β-CD inclusion complexes than that from the Ret:β-CD ones.

**Figure 7:**
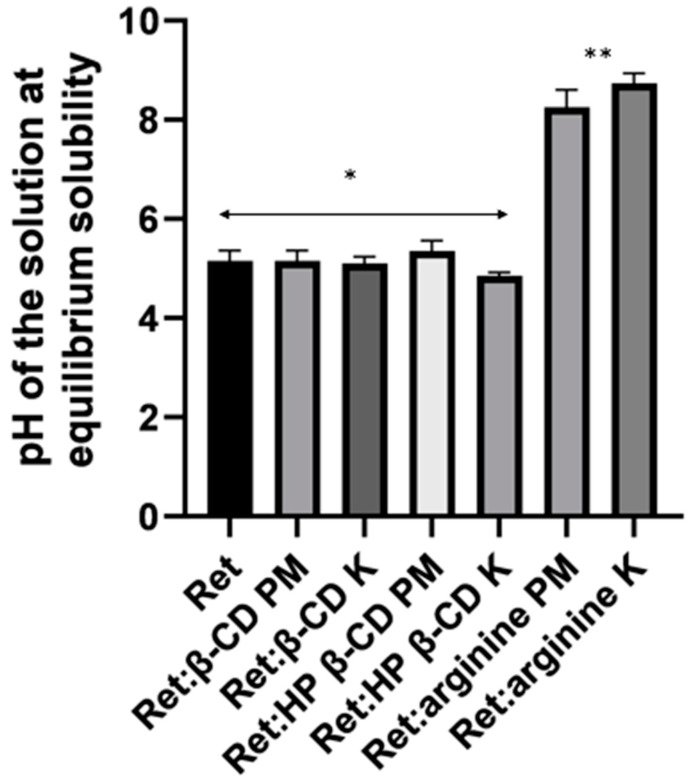
pH measurements of retinoic (Ret) alone and Ret physical (PM) and kneading (K) mixtures with the two cyclodextrins and arginine at equilibrium. Data represent means ± SD, n = 3. * Denotes non-significant differences *p* > 0.05 and ** denotes significant differences *p* < 0.05.

Regarding the Ret:arginine mixtures, the solubility of Ret was more markedly increased from both Ret:arginine PM and Ret:arginine K by 125- and 1200-folds, compared to Ret alone (Figure 6B). Arginine is a basic amino acid with a pKa of 13; this is likely to form a acid-base complex/salt with Ret and cause increased ionization and solubility of Ret. The pH of the media measured after equilibrium indicated a significant rise in pH with the Ret:arginine solution compared to those for CDs mixtures (Figure 7). Furthermore, because of the conjugation between the double bond and the nitrogen atom lone pairs, the positive charge is delocalized; thus, this could enable the formation of multiple hydrogen bonds.

### 3.3. DSC and FTIR

**Figure 8:**
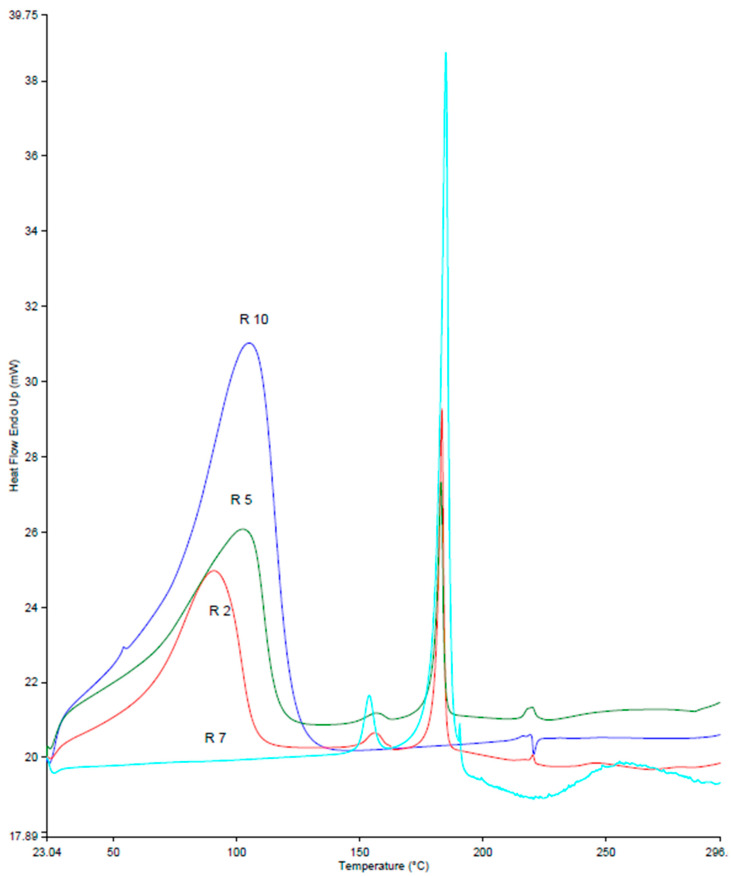
DSC thermograms for retinoic acid (Ret; R7), β-CD (R10), Ret:β-CD PM (R2), and Ret:β-CD K (R5).

Figure 8 shows the DSC thermograms of Ret, β-CD, Ret:β-CD PM, and Ret:β-CD K. Two endothermic peaks appeared at 154 and 185 °C. The weak peak at 152 °C was due to one of the polymorphic forms of Ret, and the other strong peak at 185 °C was due to the complete melting of Ret [21]. A broad peak at 105 °C was recorded for β-CD’s bound water evaporation. Both characteristic peaks of Ret and β-CD were superimposed for Ret:β-CD PM, indicating weak-to-no observable interactions recorded.

**Figure 9:**
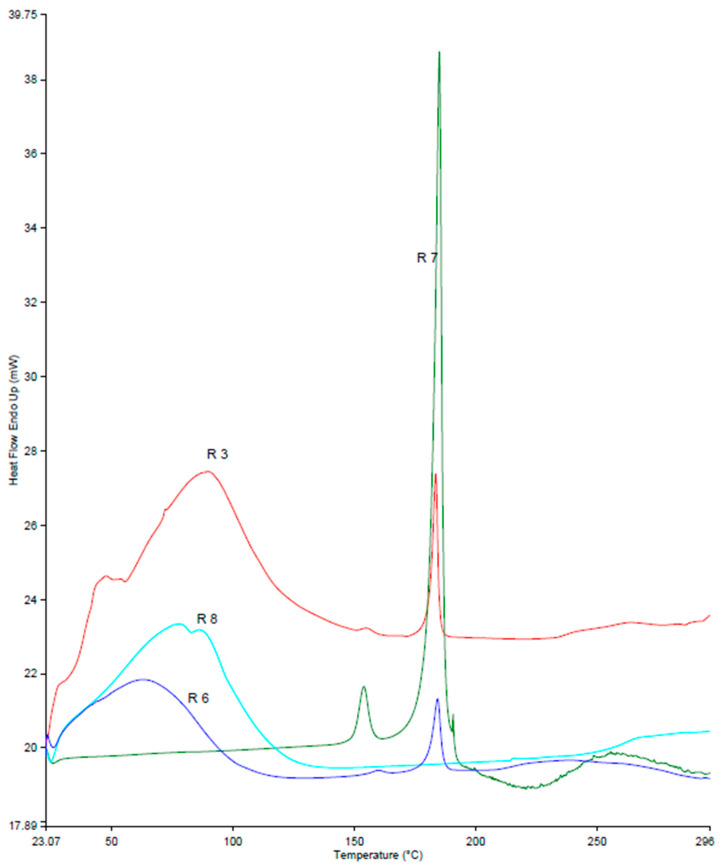
DSC thermograms for retinoic acid (Ret; R7), HP β-CD (R8), Ret:HP β-CD PM (R3), and Ret:HP β-CD K (R6).

A broad and weak endothermic peak appeared at 78 °C. This was due to surface water evaporation; see Figure 9. Similar to Ret:β-CD PM, the thermogram recorded for Ret:HP β-CD PM appeared superimposed to Ret and HP β-CD alone, indicating weak electrostatic attractions due to using the physical mixing technique. On the contrary, the appearance of drug endothermic peaks at a low intensity indicated electrostatic attractions and hydrogen bond formation with the hydroxyl groups. These results correlated well with the docking and solubility studies. Greater solubility and more favorable interactions and energy scores were recorded for the Ret:HP β-CD compared to the Ret:β-CD inclusion complexes.

**Figure 10:**
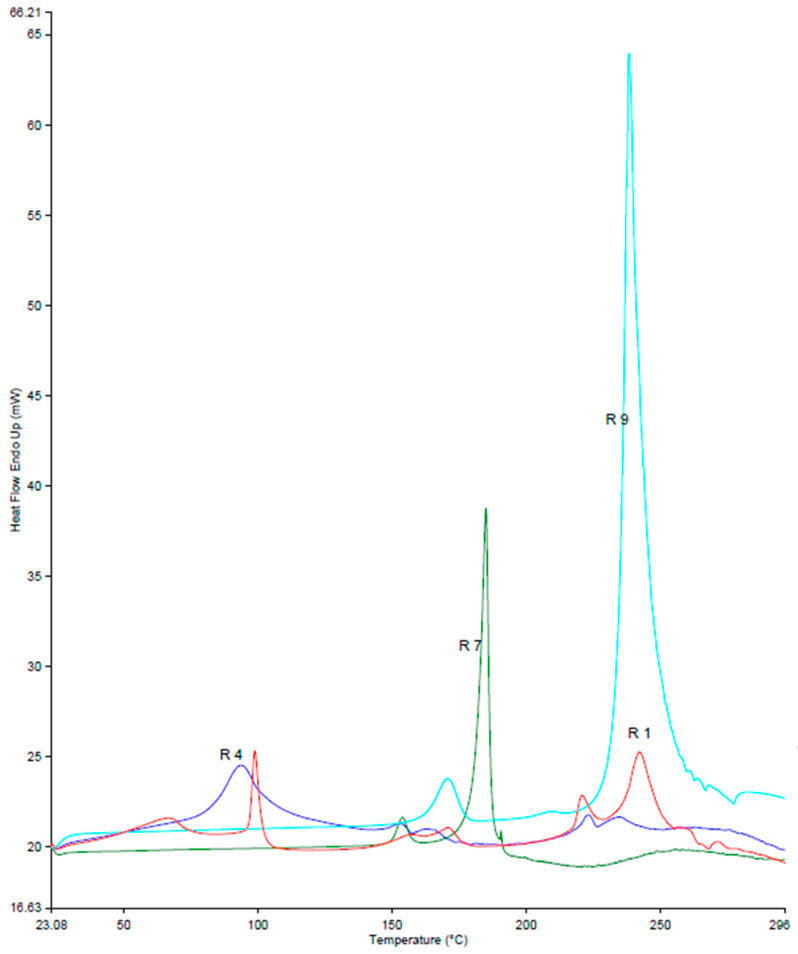
DSC thermograms for retinoic acid (Ret; R7), L-arginine (R9), Ret:arginine PM (R1), and Ret:arginine K (R4).

Figure 10 shows the DSC thermograms of Ret, arginine, Ret:arginine PM, and Ret:arginine K. Two endothermic peaks appeared at 170 and 238, which were recorded for arginine. The appearance of new peaks at 100 °C and the complete disappearance of the characteristic peaks of Ret for both Ret:arginine PM and Ret:arginine K indicate a complex/ion pair formation.

**Figure 11:**
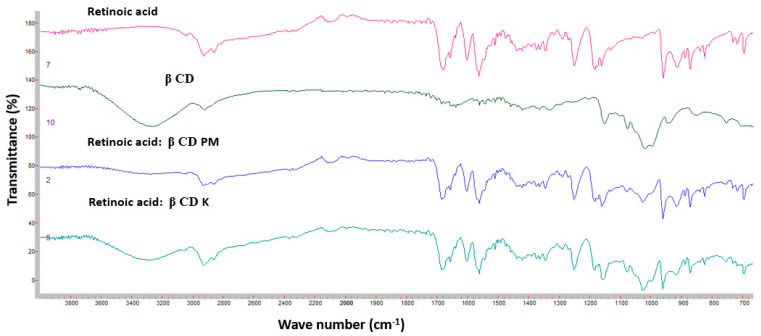
FTIR spectra of Ret, β-CD, Ret:β-CD PM, and Ret:β-CD K.

Figure 11 shows the FTIR spectra of Ret, β-CD, Ret:β-CD PM, and Ret:β-CD K. The FTIR spectrum for Ret showed medium stretching peaks at 2931 cm^–1^ and 2862 cm^–1^ for C–H alkene, 1677 cm^–1^ for C=O, and 1599 and 1560 cm^−1^ for C=C [22].

**Figure 12:**
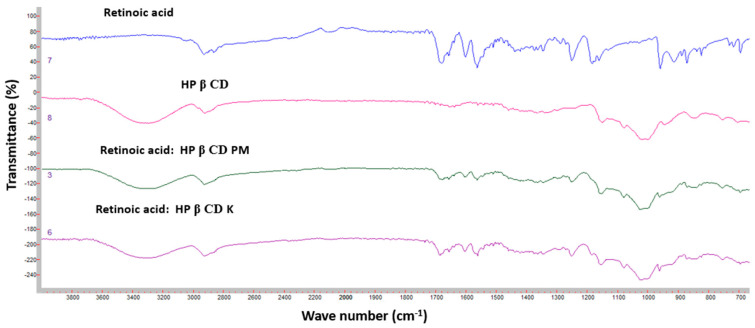
FTIR spectra of Ret, HP β-CD, Ret:HP β-CD PM, and Ret:HP β-CD K.

Broad peaks were shown at 3269 and 3254 cm^–1^ due to alcohol (–OH) stretching of the sugar molecules in β-CD and HP β-CD. The additional peak at 2924 cm^–1^ was due to C-H stretching of the cyclic structures of β-CD and HP β-CD (Figure 12). Both FTIR spectra for Ret:β-CD PM and Ret:β-CD K appeared to be superimposed for the two individual spectra of pure substances (drug and cyclodextrin), indicating weak-to-no interactions. The characteristic peaks of Ret were either shifted or broadened due to the H-bond formation.

**Figure 13:**
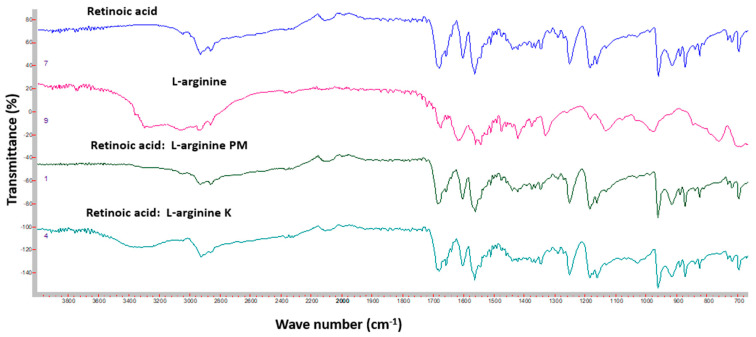
FTIR spectra of Ret, arginine, Ret:arginine PM, and Ret:arginine K.

Figure 13 shows the FTIR spectra of Ret, arginine, Ret:arginine PM, and Ret:arginine K. The FTIR spectrum for arginine showed medium stretching peaks at 3295 cm^–1^ for N–H. The FTIR spectrum for Ret:arginine PM appeared as superimposed for the Ret; an arginine individual spectrum indicated weak; undetectable-to-no interactions due to physical mixing. On the contrary, the main peaks of Ret were either shifted or broadened due to the H-bond formation for Ret:arginine K, resulting from solvent involvement and intimate contact at the molecular levels using the kneading method.

### 3.4. In Vitro Irritation Using the Cytotoxicity Assay

Skin irritation is a very common side effect of retinoic acid (Ret) topical treatment and might hamper its well-proven benefits for delaying skin aging and the treatment of acne vulgaris. The MTT assay is among the in vitro irritation models that can be used to assess the irritation potential of chemicals and is now better recognized as an alternative to the in vivo irritation assay that involves live animals and provides more quantitative endpoints [23,24]. However, the main challenge of the in vitro cytotoxicity assay is to find a relevant biological substance for skin irritants to interpret the results [24]. Therefore, azelaic acid was used as a non-to-slight irritant control with clinical relevance. Azelaic acid is commonly used for the treatment of acne vulgaris; it is applied in a massive dose of up to 20% with high skin tolerability. Azelaic acid is a natural fatty acid (1,7-hepanedicarboxylic acid). It occurs in cereals like barley, wheat, and rye. It is chemically synthetized from sunflower oil after enzymatic cleavage [25].

**Figure 14:**
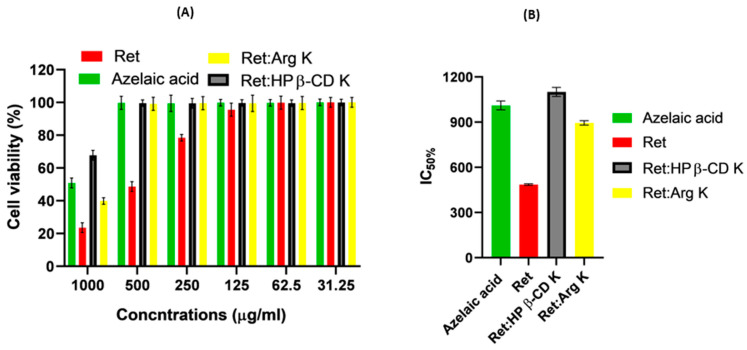
Cell viability (%) of hFB-4 cells for different concentrations of Ret, Ret:HP β-CD K, and Ret:arginine K (**A**), and IC50% of the test substances (**B**). Data represent means ± SD, n = 5.

The cell viability (%) estimated for Ret at concentrations of 500 and 1000 µg/mL were the lowest (48% and 23%, respectively) among the studied control azelaics, Ret:HP β-CD K and Ret:arginine K (Figure 14A). These results indicated that retinoic acid could pose an irritation potential when applied to the skin. Retinoic acid is available in topical creams at strengths of 0.025% to 0.1% to demonstrate that these doses are clinically relevant. On the contrary, azelaic acid recorded significantly (*p* < 0.05) higher cell viabilities (%) of 100% and 51%, respectively, at the same concentrations. More interestingly, the cell viability recorded for Ret:HP β-CD K and Ret:arginine K was significantly increased compared to that for Ret alone. The IC50% recorded for azelaic acid, Ret, Ret:HP β-CD K, and Ret:arginine K were 1000, 485, 1100, and 895 µg/mL, respectively (Figure 14B). The two carriers (HP β-CD and the amino acid arginine) were able to reduce the irritation potentials of Ret. Similar results were recorded for cyclodextrins and diclofenac sodium. The ocular irritation potential and corneal epithelial cell toxicity (%) were significantly reduced by the diclofenac/cyclodextrin inclusion complexes [26]. The inclusion of Ret in the CD pockets could reduce direct contact of the drug with the skin tissue and lessen the local high concentrations of the applied drug [2]. Similarly, the Ret/arginine ion pair complex could also neutralize high local acidity and penetration into the skin.

### 3.5. Scratch Assay

**Figure 15:**
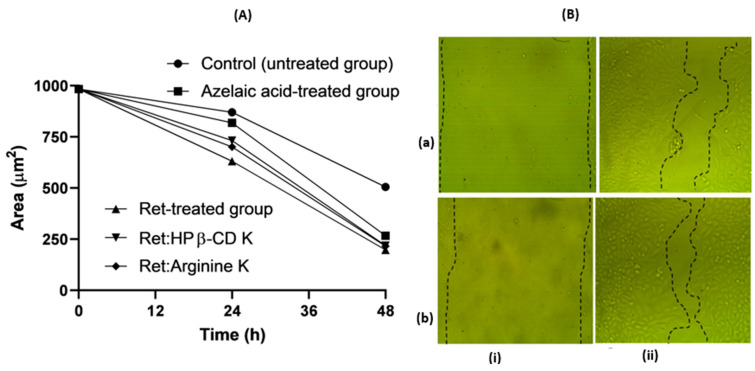
Scratch assay analysis: gap area versus time profiles for Ret and different formulations (**A**); representative micrographs (**B**) of the scratch size at zero (**i**) and 48 h (**ii**) for control (**a**) and Ret:HP β-CD K (**b**).

Vitamin A/retinoic has been reported to play an essential role in cutaneous wound healing. It promotes epithelial growth, tissue granulation, and angiogenesis [27]. Further, both topical and systemic supplements of vitamin A have proven to increase collagen deposition [28]. To evaluate the efficacy of the Ret:CD inclusion complexes and Ret/arginine ion pair, a scratch assay was performed. Ret showed a significantly (*p* < 0.05) faster gap closure compared to the untreated control (Figure 15). Comparable gap closure rates were recorded for Ret alone, Ret:HP β-CD K, and Ret:arginine K, indicating that the inclusion complexation and ion pair formation reduced the irritation potentials without undermining the efficacy.

### 3.6. Retinoic Acid-Loaded Gel Preparations

Five different gel formulations were successfully prepared from single or hybrid combinations of the cellulose (CMC), thermal gelation (Pluronic F127), and polysaccharide (gellan gum) polymers (Table 2). The prepared gels were characterized for their rheological and spreading characteristics in order to select the optimized gel formulations.

### 3.7. Viscosity and Spreading Measurements

**Figure 16:**
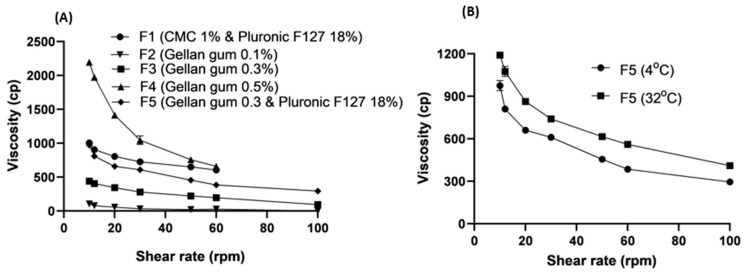
Rheological profiles of retinoic acid-based gels (**A**) for gel formulations F1–F5 and (**B**) for F5 at 4 °C and 32 °C.

Figure 16A,B shows the rheological characteristics (viscosity versus shear rate) of the prepared gels. All the prepared gel formulations showed shear-thinning (pseudoplastic flow) behaviors. However, the viscosity of the prepared gel was significantly dependent on both the concentration of gellan gum and the type of polymer. The lowest viscosity was recorded for the gellan gel (0.1%), whilst the greatest viscosity with extensive gelation was recorded for the gellan gel (0.5%). The hybrid gel combination of gellan gum 0.3% and Pluronic F127 18% (F5) showed a marked greater viscosity compared to the gellan gel alone at the same concentration. F5 was studied at two different temperatures, 4 °C (cold storage temperature) and 32 °C (skin temperature). The shear-thinning behavior of F5 remained unchanged by increasing temperature, from 4 °C to 32 °C. However, the viscosity increased with increasing the temperature, indicating thermal gelation behavior. Both F1 and F5 showed extensive gelation that can be easily handled during application on the skin. Other polymeric solutions showed runny and less viscous gels. Therefore, both F1 and F5 were considered for the spreading measurements and compared with a commercially available retinoic acid cream (Avotin-A cream).

The spreading ability of the prepared F1 and F5 gels and the commercial cream recorded 440%, 360%, and 280%, respectively. These results indicated the superior spreading ability of the prepared gels compared to the cream formulations.

### 3.8. In Vitro Release

**Figure 17:**
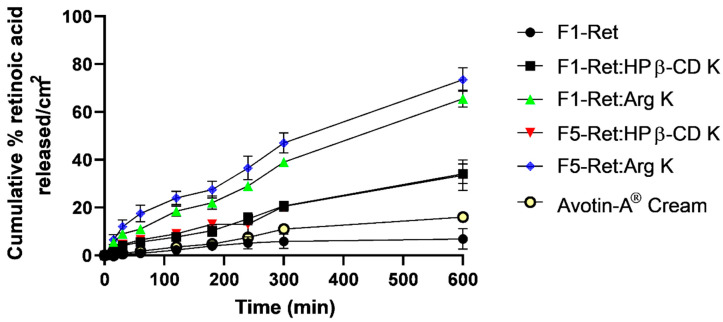
The in vitro release profiles of retinoic acid from some selected gel formulations and Avotin-A cream.

The in vitro release profiles of retinoic acid from the selected gel formulations are the F1 and F5 gels loaded with the drug alone, Ret:HP β-CD K, Ret:Arg K, and Avotin-A cream (Figure 17). The rate and extent of drug release from F1-Ret were extremely low. There are slight but not significant release rates of the drug from the cream formulation. Approximately 10% of the drug was released over 600 min and 300 min (Q10% was >600 min and 300 min) for F1-Ret and the Avotin-A cream, respectively. On the contrary, faster and greater extents were released from the Ret:HP β-CD K- and Ret:Arg K-loaded gel formulations. These results could be ascribed solely to the solubility of Ret in the tested formulations. Ret is a class II drug, and it shows solubility-dependent release rates [29]. The release rates and extents were obviously dependent on drug solubility. For example, F1-Ret showed the slowest release rate and the lowest amount released of the drug. This is because the drug was suspended in the gel matrix and more time was required for the drug to first be dissolved in the diffusion layer before its release from the polymeric matrix. On the other hand, both release rates and extents were significantly greater than the more soluble forms Ret:HP β-CD K and Ret:Arg K. More interestingly, greater dissolution rates and extents for F1-Ret:Arg K and F5-Ret:Arg K than F1-Ret:HP β-CD K and F5-Ret:HP β-CD K could be attributed to the proportional increases in solubility enhancement. The greater the solubility enhancement factor, the better the dissolution rates recorded. Both Q25% and RDR_60min_ for HP β-CD K and Ret:Arg K were recorded (600 min and 240 min) and (5.5 and 11), respectively. A slight but non-significant improvement in the dissolution rates was recorded for the Avotin-A cream compared to F1-Ret. This could be ascribed to the higher affinity of the lipophilic drug to the cream base than partitioning out to the aqueous release media. It is worth mentioning that the release rates and extent of retinoic acid of Ret:HP β-CD K and Ret:Arg K from F5 were markedly greater than those from F1. This could be attributed to the greater viscosity of F1 than F5, and hence, the time for drug diffusion out of F1 was longer than that taken to diffuse out of F5 (the less viscous polymeric matrix); see Table 3. The mechanism of drug release from the investigated systems was fitted into the Korsmeyer–Peppas equation. The release kinetic constants correlated well with the release parameters, where the lowest drug release constant was recorded for F1-Ret and the highest release constant was attributed to F5-Ret:Arg K (Table 3). The release exponent for F1-Ret:HP β-CD K and F5-Ret:HP β-CD K, and F1- and F5-Ret:Arg K as well as Ret:Arg K was 0.5 < n < 0.89, indicating a general release mechanism of coupled erosion and diffusion, while F1-Ret and the Avotin-A cream recorded n > 1, indicating a super case II-type release mechanism [30].

## 4. Conclusions

Retinoic acid (Ret) is one of the most potent and effective anti-aging and anti-wrinking cosmeceuticals. However, its irritation potential and poor solubility pose both patient compliance and formulation problems for effective long-term clinical use. Cyclodextrin and amino acids have been multifunctional excipients for improving drug solubility and tissue tolerability for many therapeutic classes. In this study, inclusion complexation with β-cyclodextrin and hydroxyl propyl β-cyclodextrin and salt formation with l-arginine were confirmed by MD, as well as thermal (DSC) and spectral (FTIR) analyses. The solubility of Ret was significantly dependent on both the method of preparation and the type of cyclodextrins. More interestingly, the solubility of Ret from Ret:arginine K increased by 1200-fold compared to Ret alone. The release rates and extent were correlated with the drug solubility from the prepared dispersion systems. Cytotoxicity was employed on the dermal cell lines as a potential in vitro irritation test. The cell viability (%) significantly increased compared to Ret alone (which posed very low cell viability). The higher the cell viability (%) recorded, the lower the irritation potential that can be interpreted. The irritation potential of Ret:CDs and Ret:arginine salt is equal to the mild-to-non-irritant control (azelaic acid) without undermining the effectiveness as a skin rejuvenating agent, as studied using the scratch assay.

## Figures and Tables

**Table 1:** The number of possible interactions and energy score (Kcal/mol) for retinoic acid docked into the inclusion pocket of β-cyclodextrin and hydroxypropyl-β-cyclodextrin.

Drug	Carrier	E Score (Kcal/mol)	Interactions	
			No. of H Bond	Bond Length (Å)
Retinoic acid	β-cyclodextrin	−5.0435	1	2.47
	Hydroxypropyl-β-cyclodextrin	−5.9529	1	1.47

**Table 2:** Codes and compositions of the prepared retinoic acid-loaded gels.

Formulation Code	Pluronic F127 (%)	CMC (%)	Gellan Gum (%)
F1	18	1	0
F2	-	-	0.1
F3	-	-	0.3
F4	-	-	0.5
F5	18	-	0.3

**Table 3:** Release parameters and kinetics of retinoic acid from two selected gel formulations (F1 and F5) loaded with drug alone, Ret:HP β-CD K and Ret:Arg K and Avotin-A cream.

Formulations	Q10% (min)	Q25% (min)	RDR_60min_	Kinetic Constant (K)	Release Exponent (n)
F1-Ret	600			0.11	1.86
F1-Ret:HP β-CD K	180	<600	5.5	0.48	0.72
F1-Ret:Arg K	60	240	11	0.67	0.65
F5-Ret:HP β-CD K	120	300	6.5	0.52	0.73
F5-Ret:Arg K	30	120	17.5	0.73	0.57
Avotin-A^®^ Cream	300		1.5	0.18	1.11

## Data Availability

The data can be shared upon request.
